# Nutrition information and the menopause: An online survey of perimenopausal and menopausal women

**DOI:** 10.1177/20533691241308370

**Published:** 2024-12-13

**Authors:** Eleanor Davies, Vanessa Halliday

**Affiliations:** 1 School of Medicine and Population Health, 7315 University of Sheffield, Sheffield, UK

**Keywords:** Menopause, postmenopause, nutrition, perimenopause

## Abstract

**Objective:**

This study aimed to explore where perimenopausal and menopausal women get their nutrition information from, and how reliable they perceive these sources to be.

**Study design:**

This was a cross-sectional study that used an online survey to collect data about nutrition information seeking habits. The survey was generated using the Qualtrics software and promoted via social media. The convenience sample included women over the age of 40 years living in the UK. Main outcome measures: Quantitative data.

**Results:**

Two hundred and sixty-nine responses were analysed. The majority of respondents were over the age of 50, and from a white British background. Online sources were most commonly used by women to access nutrition information, namely official websites such as the British Menopause Society (BMS) and National Health Service (NHS) websites. The majority of participants believed that these, along with research papers, were the most reliable sources. However, receiving nutrition information from healthcare professionals was most likely to cause women to change their behaviour towards nutrition. The majority of women were interested in receiving targeted menopausal nutrition information, and this would ideally be from official websites or their healthcare professionals.

**Conclusions:**

Findings from this study suggest that women predominantly rely on online sources for information about nutrition, perceiving official websites as reliable sources. There is a demand among women for tailored nutritional guidance during the menopausal transition, emphasising the importance of providing accessible and targeted resources to support women’s health needs during this life stage.

## Introduction

As the worldwide population ages, it is estimated that by 2030 there will be over 1.2 billion women undergoing the menopausal transition, or in the post-menopausal state.^1^ Around one third of the female population in the UK is currently perimenopausal or menopausal, and women spend more than a third of their lives in the post-menopausal state.^2,3^

Weight gain is a major consequence of the menopause, with 20% of women gaining over 4.5 kg during the perimenopausal period according to one study.^4^ The likelihood of weight gain during menopause is due to a number of factors, including change in body composition, changes to the gut microbiome, changes to sugar metabolism causing higher postprandial glucose responses, and changes in lifestyle.^3^ During the menopause, muscle mass can decrease by up to 40%, and the rate at which fat mass is gained rapidly increases.^5,6^ Over 43% of menopausal women are obese, increasing their risk of hypertension, type two diabetes and other non-communicable diseases.^3,7^ In addition, menopause and obesity both greatly increase the risk of cardiovascular disease, as well as metabolic syndrome.^6^ A recent study has found that improving the diet can help counteract the changes to metabolism that occur during the menopause, either directly by reducing blood sugar spikes and inflammation, or indirectly via the gut microbiome.^3^ Even a 5%–10% reduction in total body weight has been shown to result in many health benefits, and changes to the diet such as increased vegetable and wholegrain intake, can lead to reduced metabolic dysfunction and better health outcomes even without weight loss.^7,8^ This means that diet is a key modifiable factor in improving health outcomes in menopausal women.^3^

When it comes to finding nutrition information, evidence suggests that the landscape has largely changed over the last two decades since a landmark EU study in 1997 showing the most frequently accessed sources for information on healthy eating were TV/ radio and magazines/ newspapers.^9^ More recent data, whilst somewhat conflicting, suggests that websites or family and friends, as well as TV and news sites may now be the main sources of nutrition information.^10–12^ Social media has also been found to be a rising source of nutrition content, with nearly 70% of participants in one study claiming to use social media to access nutrition information, though most of this was accessed passively.^13^ When considering the general public’s perception of the reliability of different sources of nutrition, there are also discrepancies in the literature. The majority of studies agree that healthcare professionals (HCPs) are the most trusted source or nutrition information, though it was frequently highlighted that HCPs are often difficult to access, leading people to turn to online sources.^9,14–16^

This study aims to gain a more up to date understanding of sources of nutrition information used by the public, and how reliable these are perceived to be, targeting menopausal women who may be more likely to be searching for health and nutrition information during an important period in their lifecycle. In addition, the study will explore what topics are searched for with regards to nutrition and the menopause, to better understand what information women are interested in and if there are any key areas that could be better publicised to this population to improve their health outcomes. A final focus of this study is to explore where perimenopausal and menopausal women would prefer to obtain nutrition information from, so that recommendations can be made to help develop targeted and reliable public health nutrition messaging specific to this demographic.

## Methods

The research was conducted using a cross-sectional online survey. Convenience sampling was used with a mixture of snowballing and voluntary response sampling to recruit women over 40 years of age living in the UK. Recruitment was a multipronged approach. The survey was posted on the social media sites Twitter, LinkedIn, Instagram and Facebook by the researcher, as well as being posted into specific menopause and women’s groups on Facebook, requesting permission from group administrators. The survey was also distributed via the University of Sheffield’s volunteers list.

The survey was designed and completed anonymously using the Qualtrics software, and contained a total of 31 questions including a range of Likert scale and multiple-choice questions, as well as free text boxes where participants could give additional comments.^17^ In addition, the survey used adaptive questioning so that certain questions were conditionally displayed based on previous responses.^18^ Participant information was placed at the beginning of the survey, followed by a consent form.

The questions were developed by focussing on understanding women’s views around nutrition and the menopause and where they go to seek nutrition information. A pilot study was conducted before distribution of the survey using a sample of 10 volunteers that fit the inclusion criteria. Small adjustments were made based on feedback from the pilot, including rearranging the order of questions and changing the wording of two questions to make them clearer.

The questionnaire was made available online on 28/06/2023, accessible either via a QR code or anonymous link. The Qualtrics software ensured that participants were not able to complete the survey more than once. The survey was open for 20 days to allow for an adequate response rate. By the end of this period, responses had tailed off so it was deemed appropriate to close the survey on 24/07/2023.

### Ethical considerations and information governance

Ethical approval for this study was provided by the Sheffield Centre for Health and Related Research (SCHARR) Ethics Committee: 054030. No identifiable information was requested at any point in the survey to maintain anonymity, and high standards of information governance complying with GDPR were maintained at all times.

### Data analysis

Prior to analysis, the data was cleaned to remove any inappropriate or irrelevant responses. Descriptive statistics and content analysis of free text responses was then applied to the data.

## Results

After removing data from respondents who did not fit the inclusion criteria, a total of 269 survey responses were included in the analysis (Table 1). Of these, 23 were only partially completed, but were included as they were deemed to still contain useful information.

**Table 1.** Demographic characteristics of the women (*n* = 269) in the study.

	*n*	Frequency (%)
Age (years)		
40-44	40	14.9
45-49	57	21.2
50-54	90	33.5
55 or over	82	30.5
Country of residence		
UK	269	100
Gender identity		
Female	269	100
Ethnic group		
White/ White British	259	96.3
Black/Black British	4	1.5
Asian/ Asian British	3	1.1
Mixed ethnic background	2	0.7
Latino	1	0.4
Highest education level		
Secondary school	22	8.2
A level/ College level	45	16.8
University undergraduate	73	27.2
University postgraduate	120	44.8
Prefer not to say	1	0.4
Other	7	2.6

### Demographic information

When asked ‘Which stage do you think describes you best?’, of the four options, the majority (*n* = 119/268, 44.2%) of women were post-menopausal (not had a period for 1 year or more). One hundred and two women were self-reportedly perimenopausal (*n* = 102/268, 38%) with guidance given that this meant they were still having periods but experiencing one or more menopausal symptom. Eighteen women (*n* = 18/268, 6.7%) were not in the perimenopause/menopause, and 30/268 (11.2%) were unsure.

### Nutrition in menopause

**Figure 1.**
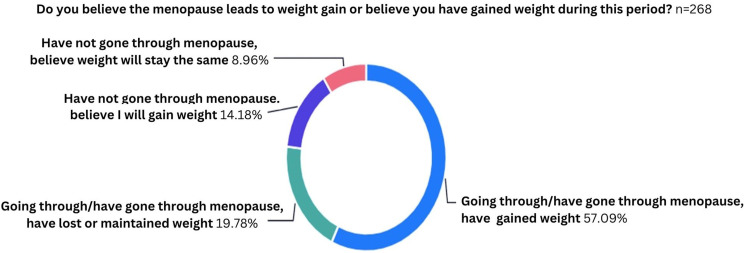
Graph to show the perceived effects of menopause on weight.

When asked about the importance of nutrition in overall health and wellbeing during the menopause, there was a mean score of 4.5 (1 = Not important at all, 5 = Very important) and the majority of women (*n* = 167/267, 63.5%) answered that it was ‘Very important’. With regards to weight gain during the menopause, 71.3% (*n* = 191/268) of women believed that they had gained weight or would do so in the future (Figure 1).

Nearly 42% (*n* = 111/266) of women also stated that they had changed what they eat due to the menopause. Content analysis of the 104 free text responses as to what dietary changes had been made found that the most common changes were reducing processed foods (*n* = 19) and eating more fruits and/or vegetables (*n* = 17). When further asked about specific diets, 61% (*n* = 164/269) were not following a specific diet, but for those that were, intermittent fasting was the most common (*n* = 36/269, 13.4%).

### Nutrition information seeking behaviour

**Figure 2.**
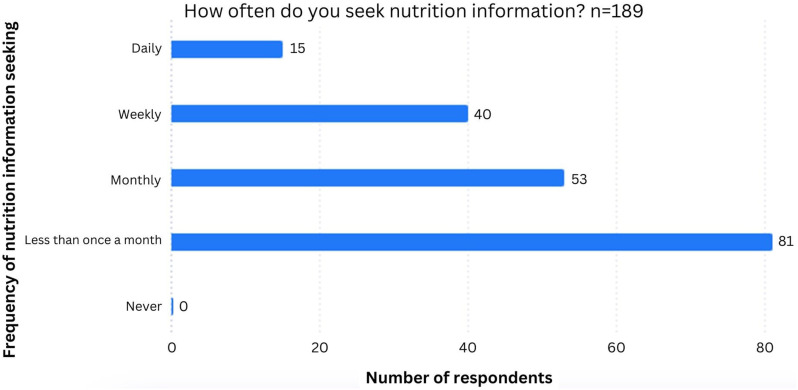
Chart to show the frequency of searching for nutrition information in participants.

When asked about their confidence in their knowledge of nutrition, women felt ‘Somewhat confident’ with an average score of 3.6 (1 = Not confident at all, 5 = Very confident). A total of 71.3% (*n* = 189/265) of participants had actively sought nutrition information at some point, and of these over half (*n* = 108/180, 57.1%) did so at least monthly (Figure 2).

When asked what specific nutrition topics were searched for, the most common topics researched included ‘Vitamins & supplements’, followed closely by ‘Weight management’, ‘Balanced diet and portion control’ and ‘managing menopausal symptoms’.

### Sources of nutrition information

Women were then asked where they accessed their nutrition information (Table 2).

**Table 2.** Table to show the most commonly accessed sources of nutrition information.

Sources of information	No. respondents	%
Official websites (e.g. NHS website, British menopause society)	168	64.4
Online articles or blogs	128	49.0
Books or magazines	108	41.4
Friends	104	39.8
Social media platforms (e.g. Facebook, Instagram, TikTok)	99	37.9
Healthcare professional (doctor, nutritionist, etc.)	81	31.0
Fitness or wellness apps	67	25.7
Television or radio	59	22.6
Research papers	48	18.4
Family	46	17.6
Weight loss groups/programme	37	14.2
Other (please specify)	30	11.5
Never accessed nutrition information	15	5.7

**Figure 3.**
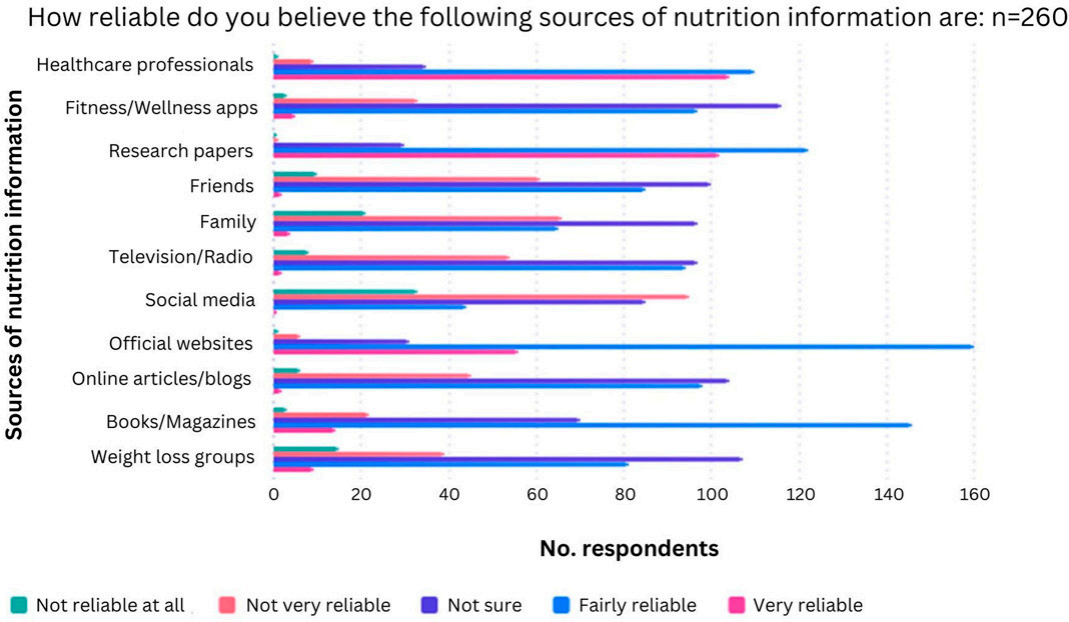
Chart to show perceived reliability of different sources of nutrition information.

The perceived most reliable sources of nutrition information appeared to be research papers followed by official websites such as the NHS website. Social media was seen as the most unreliable source, with 50% (*n* = 128/257) classing it as ‘Not very reliable’ or ‘Not reliable at all’. On the other hand, over 80% (*n* = 214/260) of participants classified healthcare professionals as either ‘Fairly reliable’ or ‘Very reliable’ (Figure 3).

When asked which (if any) of those same resources had caused them to change their behaviour towards nutrition, the most common answer was healthcare professionals (*n* = 81/247, 32.8%). Participants who had been in contact with a healthcare professional were then asked what advice they were given about nutrition and whether they followed it. The most common answer was to access a specific resource such as the NHS website or Diabetes UK, and 84.2% (*n* = 64/78) of the participants stated that they followed the advice they had been given by their healthcare professional.

In terms of usefulness in providing accessible nutrition information, official websites got the most votes for ‘Very useful’ and ‘Fairly useful’ (196/237, 82.7%). Weight loss groups were the largest group voted for ‘Not Useful at all’ (*n* = 24/244, 10.2%).

### Implications for future practice

**Figure 4.**
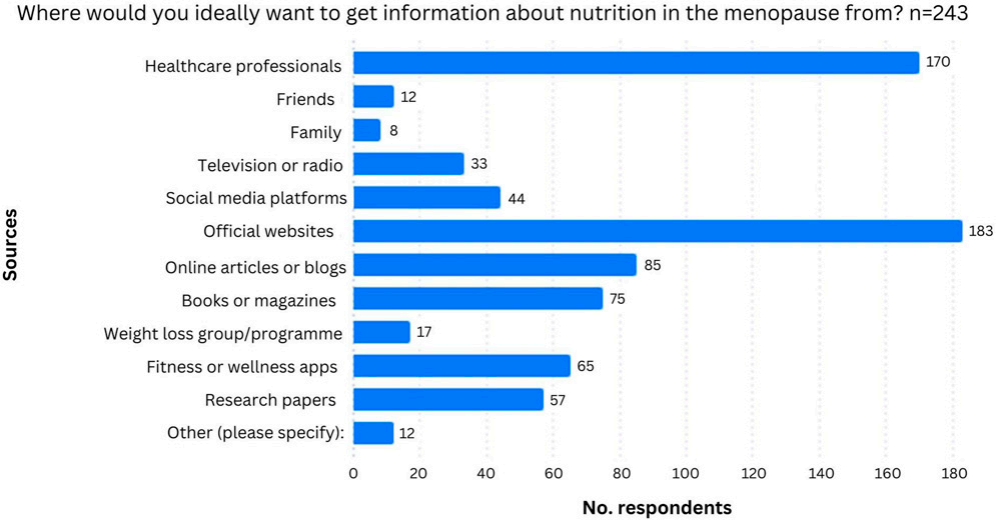
Chart to show where women would like to access information about nutrition in menopause.

The majority, (*n* = 212/244, 86.9%) of participants stated that they would be interested in receiving evidence-based nutrition information tailored specifically to menopausal women. Women were interested in getting information on a range of topics, with the most common being ‘Managing menopausal symptoms’ (*n* = 168/237, 70.9%) and ‘Bone health’ (*n* = 168/237, 70.9). The results showed that they would ideally access this information from official websites (*n* = 183/243, 75.3%) and their healthcare professionals (*n* = 170/243, 70.0%) (Figure 4).

## Discussion

The results of the survey indicate that women believe nutrition is an important consideration during the menopause, though slightly less important when it comes to the impact that nutrition might have on reducing symptoms, which is in line with the current evidence base. As with all stages of life, nutrition is important in overall health and wellbeing during the menopause, with diet a major modifiable risk factor for many non-communicable diseases.^6^ When it comes to reducing symptoms, the evidence is less clear, with some studies indicating that consumption of alcohol or high levels of fat and sugar may increase vasomotor symptoms (VMS) such as hot flushes, and a Mediterranean diet may reduce these symptoms, though the evidence is not currently strong enough to make definite recommendations.^19^ Therefore it appears from the findings of this study that women have a fairly accurate perception of the importance of nutrition during this period.

Weight gain is common during and after the menopausal transition, though the evidence as to the reason for this is conflicting, with some studies suggesting that weight gain of 0.5 kg per year is related to age rather than menopause.^20,21^ However, it has been shown that there are significant changes to metabolism and body composition during the menopausal transition, with an increase in fat mass particularly around the abdomen.^21^ This helps explain our findings that over 70% of women believed that they had or would gain weight during the menopause. However, research shows that this does not have to be inevitable, and that changes to the diet and exercise can help slow or prevent weight gain during and after menopause, though these interventions are most effective if undertaken during the perimenopausal period.^22^

Nearly 58% of participants in the current study stated that they had not changed what they eat due to the menopause. However, over 70% of the respondents had actively sought out nutrition information, and of these the majority did so once a month or more. Whilst there are currently no studies looking into the matter, it may also be assumed that with the rise of Internet access and social media, passive access to nutrition information is also on the rise, so that women may have even seen nutrition information more frequently than this. Women were most interested in information about vitamins and supplements, which are often seen as a ‘quick fix’, but may actually be useful to women during this period according to some studies, though the evidence is inconsistent.^19^ Weight management was another key search topic, which fits with the fact that women are likely to gain weight during this period and therefore want to find effective ways to lose it. Women stated that they were slightly more likely to be researching these topics due to the menopause, though due to the study sample it is feasible to conjecture that women may have been researching nutrition despite menopause.

The primary objective of this study was to identify the key sources used by women over 40 to find information about nutrition. It was found that the most commonly used resources were official websites, followed by online articles or blogs. Online resources were therefore clearly the preferred method, though this may have been heavily influenced by the demographic of women undertaking the survey, many of whom were recruited via online channels and so had a higher propensity to use online resources. However, this strengthens the idea that the Internet is a key place where people access health information, and should therefore be utilised by those trying to spread accurate health information to this demographic.

This finding is in line with other studies that found online sources to be most popular, such as a study in Ghana where 92.7% of the sample had used the Internet to seek nutrition information, though this was in a sample of 18–25 year olds.^10^ Similarly, in the USA, 78% of students in one study had looked online for health information, and another found websites to be the most popular source of nutrition information (58.6%) in a survey of 476 lay people.^23,24^ Although there is a large diversity in the study populations, it is clear that the Internet is an increasingly popular source of health and nutrition information, and so it is essential that available information is accurate and informative.

This research offers new insights into the perceived reliability of different sources of information. Although this study found that participants believed research papers were the most reliable source of nutrition information, this differs from research done by the Academy of Medical Sciences where people were more likely to trust the experiences of their friends and family than data from clinical trials.^25^ In our study, official websites were found to be the second most reliable source, in line with other studies that found that online sources are generally perceived as reliable.^10,14,26^ These websites were also seen as the most useful by participants, which may explain why 63% of the UK population went online to seek health information in 2020, and the NHS website has more than 50 million visits every month.^27,28^ On the other hand, social media was seen as the least reliable source of nutrition information by participants of the survey. This may be a reasonable perception given that other studies have found large amounts of inaccurate information about nutrition on social media, particular surrounding diet misinformation.^29^ In addition, healthcare professionals were only accessed by 31% of the study population, but were seen as reliable by over three quarters of the sample. This agrees with other studies that despite healthcare professionals being seen as one of the most reliable sources, they are not commonly accessed.^10^ However, despite the fact they are seen as reliable and are the most likely source to enforce behaviour change, doctors themselves do not feel that they receive sufficient nutrition education.^30^ A survey of junior doctors found that 91% would like to receive more teaching on nutrition as part of medical training, and despite them being aware that patients expect them to have a thorough understanding, only a quarter (26%) felt confident discussing nutrition with patients.^30^

The women in our sample were interested in receiving evidence-based nutrition information specific to menopause on a range of topics including ‘managing menopausal symptoms’ and ’bone health’. Findings indicated that women would ideally like to find targeted nutrition information on official websites, as it is clear these are more commonly accessed and are seen as reliable. Looking at the current information available on official websites, the NHS website currently recommends a healthy diet, including sources of calcium and taking vitamin D supplements, as ways to protect against weak bones during menopause. However, this is all the nutrition information provided and it does not have a specific page concerning nutrition and the menopause.^31^ On the other hand, the British Nutrition Foundation have a dedicated page to nutrition and menopause, where key information women were interested in in this study can be found.^32^ However, women, particularly those without a specific interest in nutrition, may not know where and how to look for this information. Therefore, in order to improve public health nutrition messaging, information such as that on the British Nutrition Foundation site should be publicised further on other official websites, and on other platforms such as social media to ensure a larger number of women are accessing this information. In addition, as doctors are seen as one as the most reliable sources of nutrition information, and they are the most likely to help patients change their behaviour, it is clear that more consideration must be taken in the training of doctors with regards to nutrition to ensure patients are receiving the most effective care.

## Limitations

There are several limitations that should be taken into account when interpreting the findings of this study. As with any survey, this study is limited to the sample population, of whom the majority were of White British ethnicity and were educated to postgraduate level, compromising the generalisability of the study. The survey also excluded women under 40 who may be going through premature menopause.

Due to the non-random nature of sampling, there may have been significant amounts of bias in the survey responses. The majority of participants were recruited voluntarily via menopause Facebook groups, thereby indicating that they already have an interest in learning more about menopause, and therefore may have been more motivated to answer, introducing selective non-response bias.

## Conclusion

Overall, this study provides valuable information to help guide healthcare practitioners, policymakers and other interested parties about the best places to disseminate nutrition information for perimenopausal and menopausal women. Ensuring women have access to all the information they need during what can be a difficult period in their lives is essential so that they can feel fully informed and prepared to make the best choices for their health and wellbeing.
